# Association of Economic Status and Mortality in Patients with Acute Respiratory Distress Syndrome

**DOI:** 10.3390/ijerph17061815

**Published:** 2020-03-11

**Authors:** Tak Kyu Oh, In-Ae Song, Jae Ho Lee

**Affiliations:** 1 Department of Anesthesiology and Pain Medicine, Seoul National University Bundang Hospital, Seongnam 13620, Korea; airohtak@hotmail.com; 2 Division of Pulmonary and Critical Care Medicine, Department of Internal Medicine, Seoul National University Bundang Hospital, Seongnam 13620, Korea; jhlee7@snubh.org

**Keywords:** insurance, respiratory distress syndrome, adult, economic status, critical care

## Abstract

The high cost of treatment for acute respiratory distress syndrome (ARDS) is a concern for healthcare systems, while the impact of patients’ socio-economic status on the risk of ARDS-associated mortality remains controversial. This study investigated associations between patients’ income at the time of ARDS diagnosis and ARDS-specific mortality rate after treatment initiation. Data from records provided by the National Health Insurance Service of South Korea were used. Adult patients admitted for ARDS treatment from 2013 to 2017 were included in the study. Patients’ income in the year of diagnosis was evaluated. A total of 14,600 ARDS cases were included in the analysis. The 30-day and 1-year mortality rates were 48.6% and 70.3%, respectively. In multivariable Cox regression model, we compared income quartiles, showing that compared to income strata Q1, the Q2 (*p* = 0.719), Q3 (*p* = 0.946), and Q4 (*p* = 0.542) groups of income level did not affect the risk of 30-day mortality, respectively. Additionally, compared to income strata Q1, the Q2 (*p* = 0.762), Q3 (*p* = 0.420), and Q4 (*p* = 0.189) strata did not affect the risk of 1-year mortality. Patient income at the time of ARDS diagnosis did not affect the risk of 30-day or 1-year mortality in the present study based on South Korea’s health insurance data.

## 1. Introduction

Acute respiratory distress syndrome (ARDS) is defined as respiratory failure caused by an acute onset of hypoxemia and bilateral pulmonary infiltrates [1,2,3]. The incidence of ARDS among patients in the intensive care unit (ICU) is 10.4% [4], and associated mortality has been reported as 11–87% [5]. According to previous reports, the global burden of ARDS is highest in high- and upper-middle-income countries; the syndrome is an important global health issue [6,7].

Given the high mortality risk associated with ARDS, treatment for the disease requires an ICU admission, resulting in an average daily cost of $2100 per patient, and mean total annual cost at 1 year of $53,300 [8]. Previous studies have shown that patients’ economic status might affect their mortality risk within an ICU [9,10]; it follows that the risk of ARDS-specific mortality might be influenced by each patient’s income. In fact, previous studies have reported that economic status might affect mortality risk associated with chronic lung diseases such as asthma [11] and cystic fibrosis [12]. Moreover, Bime et al. showed that household income reported for patients’ ZIP codes was associated with in-hospital mortality due to severe acute respiratory failure in the United States [13]. Furthermore, a prospective cohort study from the LUNG SAFE trial has shown that income per person was associated with ARDS outcomes [14]. However, these findings might be mediated by the structure of each country’s health insurance system; further research is required to elucidate the link between patients’ income and the risk of ARDS-associated mortality. In South Korea, individuals are generally registered in the National Health Insurance Service (NHIS) as a requirement and income levels of all individuals are recorded accurately on the first day of each month to determine insurance fees for all individuals [15].

This study aimed to investigate any association between the income level of ARDS patients at the time of diagnosis and their mortality risk after initiation of ARDS treatment. We hypothesized that a lower income level might be associated with a higher mortality risk among ARDS patients.

## 2. Materials and Methods

### 2.1. Ethical Statement

All procedures performed on human participants were in accordance with the ethical standards of the institutional and/or national research committee (Seoul National University Bundang Hospital (X-1903-531-902) and the Health Insurance Review and Assessment Service (NHIS-2019-1-274).

### 2.2. Data Source

We used health records from the national database of South Korea, provided by the National Health Insurance Service (NHIS). The NHIS, which is the nation’s key health-protecting institution, is responsible for the management of national health insurance, which provides disease-related and long-term care, ensuring comfortable aging. In South Korea, data on all diagnoses of disease alongside prescription information (including drugs and procedures) are included in the NHIS database. Additionally, the population’s annual income level is registered by the NHIS, as it is used to determine insurance fees. However, despite differences in fees, Koreans tend to receive similar coverage from the NHIS, which is equivalent to approximately 2/3’s of the co-payment for total hospital charges. Furthermore, if patients cannot afford their insurance premium or face financial difficulties, the government covers almost all their medical expenses. For the purpose of this study, data extraction was performed by a medical records technician at the NHIS center.

### 2.3. Study Population

All adult patients (≥18 years old) admitted to a hospital for ARDS-related treatment (International Classification of Diseases; ICD-10 codes of J80*) from 1 January 2013, to 31 December 2017, were included in this study. In cases of multiple admissions (≥2) for ARDS-related treatment per patient, only the most recent admission was included in the analysis. Additionally, cases with incomplete or missing data on income level were excluded.

### 2.4. Income Level as An Independent Variable

Using the NHIS database, income levels of ARDS patients in the year of diagnosis and treatment were evaluated. Annual income of patients at the time of diagnosis of ARDS was divided into four groups (Q1–Q4), using quartile ratios.

### 2.5. Study Endpoint

The primary endpoint was the 30-day or 1-year mortality rate, following initiation of ARDS treatment. Mortality data were extracted until 30 May 2019. Survival time was calculated from the date of initial ARDS treatment to the date of death, or 30 May 2019, for ARDS survivors.

### 2.6. Confounders

The data collected as confounders for this study included demographic characteristics (age and gender) and place of residence at the time of diagnosis of ARDS (Seoul, metropolitan cities, and other). In the classification of residence, Seoul, the capital city, was assigned a separate category, and the cities of Incheon, Kwangju, Busan, Ulsan, Daegu, and Daejeon were classified as metropolitan cities. Comorbidities before diagnosis of ARDS were considered under corresponding ICD-10 codes (hypertension (I10–I16), diabetes mellitus (E10–E14), coronary artery disease (I20*–I25*), cerebrovascular disease (I60*–I69*), lung cancer (C30–C39), chronic kidney disease (N18*), dyslipidemia (E78.0), anemia (D64*), chronic obstructive lung disease (J44*), asthma (J45*), arrhythmia (I49*), and liver cirrhosis (K74*)). Extracorporeal membrane oxygenation (ECMO), as part of ARDS treatment, was also considered.

### 2.7. Statistical Analysis

Baseline characteristics of the ARDS patients were presented as means and standard deviations (SD) for continuous variables and counts with percentages for categorical variables. We performed a multivariable Cox regression analysis for two dependent variables (30-day mortality and 1-year mortality). All covariates (age, gender, place of residence, and comorbidities before ARDS diagnosis) were included in these multivariable models. It was confirmed that there was no multi-collinearity in any multivariable model with variance inflation factors <2.0, and a log–log plot was used to confirm that the central assumption of each multivariable model was satisfied. In addition, the C-statistic was used to investigate the goodness-of-fit of the multivariable models for predicting 30-day and 1-year mortality, following ARDS treatment; these findings were reported as a C-index with the corresponding 95% confidence interval (CI). The Cox regression model was presented as a hazard ratio (HR) with 95% CIs. Finally, a survival plot derived from the multivariable Cox regression model for 1-year mortality was presented according to the four groups of income levels. All analyses were performed using SAS software version 9.4 (SAS Institute Inc., Cary, NC, USA), and a *p* < 0.05 was considered statistically significant.

## 3. Results

### 3.1. Patients

**Figure 1:**
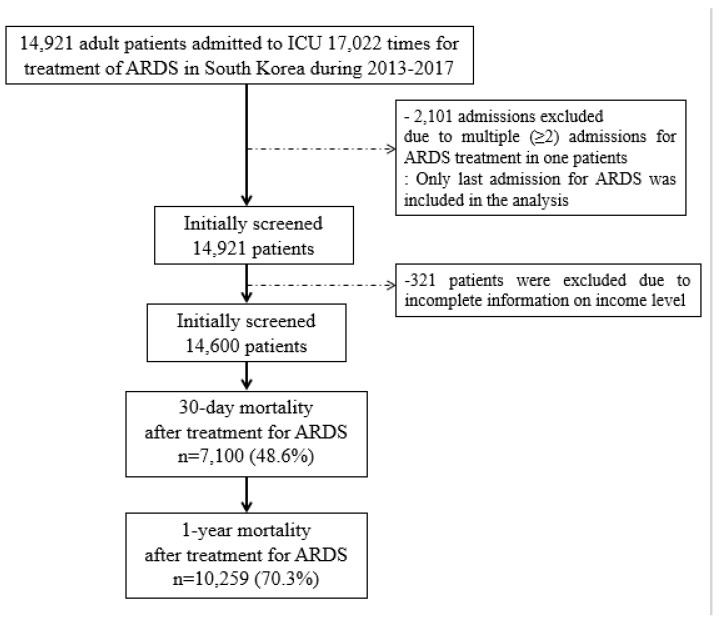
Flow chart depicting patient selection. ARDS, acute respiratory distress syndrome.

From 1 January 2013, to 31 December 2017, 14,921 adult patients were admitted to the ICU 17,022 times for treatment of ARDS in South Korea. Among them, 2101 admissions were excluded, as they were multiple (≥2) admissions of the same patient. Of the included patients, 321 patients were excluded due to incomplete income information at diagnosis of ARDS. Finally, 14,600 ARDS patients were included in the analysis. Among them, 7100 patients (48.6%) and 10,259 patients (70.3%) died within 30-days and 1-year after initiation of ARDS treatment, respectively (Figure 1). The baseline characteristics of South Korean ARDS patients are presented in Table 1. The mean length of hospital stay among these patients was 16.2 days (SD: 15.0 days), and the mean duration of treatment was 18.4 days (SD: 17.1 days). Meanwhile, 681 patients (4.7%) were treated using ECMO.

### 3.2. Survival Analysis According to Income Level

**Figure 2:**
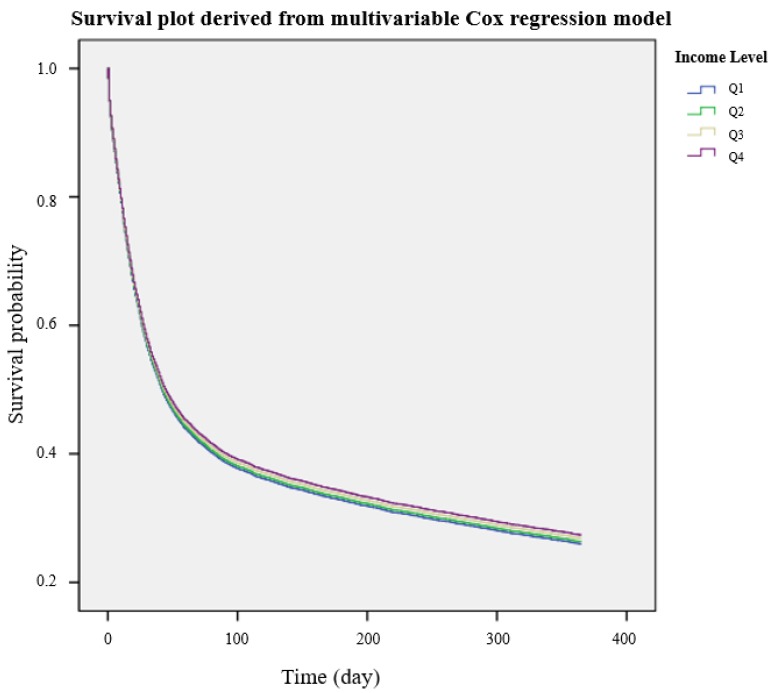
Survival among patients diagnosed with ARDS, based on their income level in the year preceding the diagnosis (estimates derived from the multivariable Cox regression model). ARDS, acute respiratory distress syndrome.

Table 2 shows the results of the multivariable Cox regression models for 30-day and 1-year mortality in the ARDS patients according to income level. Using Q1 income level as a reference, Q2 (HR = 1.02, 95% CI: 0.94–1.10; *p* = 0.719), Q3 (HR = 1.00, 95% CI: 0.93–1.08; *p* = 0.946), and Q4 (HR = 0.98, 95% CI:0.91–1.05; *p* = 0.542) income levels were not associated with the risk of 30-day mortality. Similarly, with the same reference, income levels Q2 (HR = 0.99, 95% CI: 0.93–1.06; *p* = 0.762), Q3 (HR = 0.98, 95% CI: 0.92–1.04; *p* = 0.420), and Q4 (HR = 0.96, 95% CI: 0.91–1.02; *p* = 0.189) were not associated with the risk of 1-year mortality. The C-index for the 30-day mortality was 0.76 (95% CI: 0.75–0.77), and for the 1-year mortality it was 0.90 (95% CI: 0.89–0.91). The survival plot derived from the multivariable Cox regression model is presented in Figure 2, showing a similar survival trend in the 20 strata of income.

## 4. Discussion

This nationwide cohort study from South Korea has shown that income level at diagnosis of ARDS was not significantly associated with the risk of 30-day or 1-year mortality following initiation of ARDS treatment. Our results suggest that economic status prior to diagnosis of ARDS is not a significant factor when considering the prognosis of ARDS patients treated within the NHIS system of South Korea. These findings are important, as the NHIS system in South Korea covers 2/3’s of the total medical costs for the general population [15], increasing the coverage to 100% for groups that require financial support [16]. In contrast to previous studies reporting on the impact of individuals’ income on their ARDS-related prognosis within different health insurance systems [13,14], we present evidence that income might not be an important prognostic factor for ARDS patients, given suitable public health insurance coverage.

Our results are different from those of a similar previous study by Bime et al. [13], which has shown that median household income reported for patients’ ZIP codes was associated with in-hospital mortality in patients with severe acute respiratory failure. The study by Bime and colleagues analyzed data from patients with severe acute respiratory failure diagnosed during 2008–2012, using the ICD-9 diagnostic system; in contrast, our study used data from patients with ARDS diagnosed during 2013–2017, using the ICD-10 diagnostic system. Since the Berlin definition of ARDS was developed in 2012 by the ARDS task force [3], the ARDS patients in our nationwide study might have been diagnosed based on different criteria compared with the study by Bime et al. Considering that the incidence of ARDS might be underestimated when based on the ICD-9 diagnostic system [17], our results are likely more reliable than the previously reported results. Furthermore, the accuracy of income level data in our study is higher, as data on income levels of all ARDS patients were extracted from the NHIS database.

The results in our study might be influenced by ARDS being the most fatal clinical syndrome. Since the mortality of ARDS has been reported as 87% in previous studies [5], the impact of economic status on mortality risk might be limited. Furthermore, most critically ill patients in South Korea might be treated with a similar level of care, as 2/3’s of treatment costs are covered by the NHIS [15]. Therefore, the results in this study might differ from results reported for countries where the government does not pay for healthcare to a similar extent. While evidence is insufficient regarding the association between income, insurance status, and ARDS-related mortality in other countries, previous studies have shown that a lack of insurance is associated with a higher mortality rate among critically ill patients with sepsis [18,19], which is a comparably critical illness. Therefore, more studies are needed to confirm the association between economic status and ARDS-specific mortality risk in different insurance settings.

The present study reported on 30-day and 1-year mortality, because we had survival time data for all ARDS patients followed-up for at least 365 days. The 1-year mortality rate among ARDS patients might be a measure of long-term outcomes. Previous studies have reported that ARDS survivors commonly suffer from persistent functional disability 1 to 5 years after ICU discharge [20,21]. Recovery after ICU discharge among ARDS patients is an important challenge [22]. In fact, studies have shown that most ARDS survivors have lost their previous job and never resume employment post-discharge [23,24]. Critically ill patients in the United States who do not have health insurance receive fewer critical care services and may experience worse clinical outcomes [25]. From these perspectives, our study has shown that financial support provided by the government as national insurance reduces the impact of personal income on relatively long-term mortality. Financial support might be needed for ARDS patients with a lower economic status to ensure their health prognoses are similar to the prognoses of people within higher economic strata.

This study has several limitations. First, some important information, such as body mass index, was not included in the analysis because the NHIS database does not provide this information. Second, we used the ICD-10 codes registered in the NHIS database to define comorbidities. The diseases specified in the ICD-10 codes may have differed from the actual underlying diseases. Third, there might be some ARDS patients not registered as having ARDS under the ICD-10 diagnostic system (J80*). Fourth, we did not consider some important characteristics of ARDS patients, such as the P/F ratio, simplified acute physiology score-II, and the acute physiology and chronic health evaluation II score, which are closely related to patient prognosis; these data were not available from the NHIS database. Lastly, since this study was based on a retrospectively collected dataset, there might be residual confounders that might affect the results of this study.

## 5. Conclusions

This nationwide cohort study showed that income level before ARDS diagnosis was not significantly associated with 30-day and 1-year mortality among ARDS patients captured by the NHIS coverage system in South Korea. These findings suggest that individuals’ economic status might not be an important prognostic factor for ARDS, given suitable public health insurance coverage. Well-designed and prospective cohort studies should be performed to confirm these findings in other countries with different insurance systems.

## Figures and Tables

**Table 1:** Baseline characteristics of acute respiratory distress syndrome (ARDS) patients in South Korea from 2013 to 2017.

Variable		Total (14,600), n (%)	Mean (SD)
Age, year			69.8 (15.3)
Gender, male		9065 (62.1)	
Residence at diagnosis			
	Capital city, Seoul	2402 (16.5)	
	Other metropolitan city ^a^	3334 (22.8)	
	Others	8864 (60.7)	
Income level at diagnosis			
	Q1	2449 (16.8)	
	Q2	2732 (18.7)	
	Q3	4199 (28.8)	
	Q4	5220 (35.8)	
Length of hospital stay, day			16.2 (15.0)
Duration of treatment, day			18.4 (17.1)
ECMO use for treatment		681 (4.7)	
Comorbidities before diagnosis			
	Hypertension	9735 (66.7)	
	Coronary artery disease	4403 (30.2)	
	Diabetes mellitus	6500 (44.5)	
	Cerebrovascular disease	5044 (34.5)	
	Lung cancer	1125 (7.7)	
	Chronic kidney disease	1434 (9.8)	
	Dyslipidemia	8021 (54.9)	
	Anemia	2733 (18.7)	
	COPD	3819 (26.2)	
	Asthma	1809 (12.4)	
	Arrhythmia	1017 (7.0)	
	Liver cirrhosis	408 (2.8)	
Diagnosis per year			
	2013	1978 (13.5)	
	2014	2089 (14.3)	
	2015	2065 (14.1)	
	2016	2289 (15.7)	
	2017	6179 (42.3)	

Metropolitan city ^a^ include Incheon, Kwangju, Busan, Ulsan, Daegu, and Daejeon. ARDS, acute respiratory distress syndrome; ECMO, extracorporeal membrane oxygenation; COPD, chronic obstructive pulmonary disease.

**Table 2:** Multivariable Cox regression model for 30-day and 1-year mortality according to income level.

Variable		30-Day Mortality	*p*	1-Year Mortality	*p*
		HR (95% CI)		HR (95% CI)	
Income level at ARDS diagnosis					
	Q1	1		1	
	Q2	1.02 (0.94, 1.10)	0.719	0.99 (0.93, 1.06)	0.762
	Q3	1.00 (0.93, 1.08)	0.946	0.98 (0.92, 1.04)	0.420
	Q4	0.98 (0.91, 1.05)	0.542	0.96 (0.91, 1.02)	0.189
Age, year		1.02 (1.01, 1.02)	<0.001	1.02 (1.01, 1.02)	<0.001
Gender, male		1.00 (0.95, 1.05)	0.895	1.03 (0.99, 1.07)	0.167
Residence at diagnosis					
	Capital city, Seoul	1		1	
	Other metropolitan city	1.18 (1.09, 1.27)	<0.001	1.12 (1.06, 1.20)	<0.001
	Others	1.11 (1.04, 1.19)	0.002	1.04 (0.98, 1.09)	0.211
ECMO use for treatment		1.30 (1.16, 1.45)	<0.001	1.38 (1.25, 1.52)	<0.001
Comorbidity before diagnosis					
	Hypertension	0.92 (0.87, 0.97)	0.004	0.93 (0.89, 0.97)	0.002
	Coronary artery disease	0.97 (0.92, 1.03)	0.285	0.95 (0.91, 1.00)	0.029
	Diabetes mellitus	0.99 (0.94, 1.04)	0.687	0.98 (0.94, 1.02)	0.397
	Cerebrovascular disease	0.89 (0.84, 0.94)	<0.001	0.93 (0.89, 0.97)	0.002
	Lung cancer	0.99 (0.91, 1.08)	0.799	1.09 (1.02, 1.17)	0.017
	Chronic kidney disease	0.80 (0.73, 0.87)	<0.001	0.84 (0.78, 0.90)	<0.001
	Dyslipidemia	1.06 (1.00, 1.12)	0.039	1.07 (1.02, 1.12)	0.004
	Anemia	0.94 (0.88, 1.00)	0.050	0.95 (0.87, 1.03)	0.182
	COPD	0.76 (0.71, 0.80)	<0.001	0.77 (0.74, 0.81)	<0.001
	Asthma	0.98 (0.90, 1.06)	0.539	0.96 (0.90, 1.02)	0.194
	Arrhythmia	0.95 (0.86, 1.05)	0.332	0.95 (0.87, 1.03)	0.182
	Liver cirrhosis	1.17 (1.01, 1.34)	0.034	1.17 (1.04, 1.31)	0.010
Diagnosis per year					
	2013	1		1	
	2014	0.86 (0.80, 0.93)	<0.001	0.84 (0.78, 0.89)	<0.001
	2015	0.84 (0.78, 0.91)	<0.001	0.76 (0.72, 0.82)	<0.001
	2016	0.79 (0.73, 0.85)	<0.001	0.70 (0.66, 0.75)	<0.001
	2017	0.25 (0.23, 0.27)	<0.001	0.16 (0.15, 0.18)	<0.001

C-index for 30-day mortality: 0.76 (95% CI: 0.75–0.77); and for 1-year mortality: 0.90 (95% CI: 0.89–0.91). HR, hazard ratio; CI, confidence interval; ECMO, extracorporeal membrane oxygenation; COPD, chronic obstructive pulmonary disease.

## References

[1] Ashbaugh D.G., Bigelow D.B., Petty T.L., Levine B.E. (1967). Acute respiratory distress in adults. Lancet.

[2] Bernard G.R., Artigas A., Brigham K.L., Carlet J., Falke K., Hudson L., Lamy M., Legall J.R., Morris A., Spragg R. (1994). The american-european consensus conference on ards. Definitions, mechanisms, relevant outcomes, and clinical trial coordination. Am. J. Respir. Crit. Care Med..

[3] Force A.D.T., Ranieri V.M., Rubenfeld G.D., Thompson B.T., Ferguson N.D., Caldwell E., Fan E., Camporota L., Slutsky A.S. (2012). Acute respiratory distress syndrome: The berlin definition. JAMA.

[4] Bellani G., Laffey J.G., Pham T., Fan E., Brochard L., Esteban A., Gattinoni L., Van Haren F., Larsson A., McAuley D.F. (2016). Epidemiology, patterns of care, and mortality for patients with acute respiratory distress syndrome in intensive care units in 50 countries. JAMA.

[5] Maca J., Jor O., Holub M., Sklienka P., Bursa F., Burda M., Janout V., Sevcik P. (2017). Past and present ards mortality rates: A systematic review. Respir. Care.

[6] Adhikari N.K., Fowler R.A., Bhagwanjee S., Rubenfeld G.D. (2010). Critical care and the global burden of critical illness in adults. Lancet.

[7] Riviello E.D., Kiviri W., Twagirumugabe T., Mueller A., Banner-Goodspeed V.M., Officer L., Novack V., Mutumwinka M., Talmor D.S., Fowler R.A. (2016). Hospital incidence and outcomes of the acute respiratory distress syndrome using the kigali modification of the berlin definition. Am. J. Respir. Crit. Care Med..

[8] Marti J., Hall P., Hamilton P., Lamb S., McCabe C., Lall R., Darbyshire J., Young D., Hulme C. (2016). One-year resource utilisation, costs and quality of life in patients with acute respiratory distress syndrome (ards): Secondary analysis of a randomised controlled trial. J. Intensive Care.

[9] Oh T.K., Jo J., Jeon Y.-T., Song I. (2018). Impact of socioeconomic status on 30-day and 1-year mortalities after intensive care unit admission in south korea: A retrospective cohort study. Acute Crit. Care.

[10] Schnegelsberg A., Mackenhauer J., Nibro H.L., Dreyer P., Koch K., Kirkegaard H. (2016). Impact of socioeconomic status on mortality and unplanned readmission in septic intensive care unit patients. Acta Anaesthesiol. Scand..

[11] Sinharoy A., Mitra S., Mondal P. (2018). Socioeconomic and environmental predictors of asthma-related mortality. J. Environ. Public Health.

[12] Schechter M.S., Shelton B.J., Margolis P.A., Fitzsimmons S.C. (2001). The association of socioeconomic status with outcomes in cystic fibrosis patients in the united states. Am. J. Respir. Crit. Care Med..

[13] Bime C., Poongkunran C., Borgstrom M., Natt B., Desai H., Parthasarathy S., Garcia J.G. (2016). Racial differences in mortality from severe acute respiratory failure in the united states, 2008–2012. Ann. Am. Thorac. Soc..

[14] Laffey J.G., Madotto F., Bellani G., Pham T., Fan E., Brochard L., Amin P., Arabi Y., Bajwa E.K., Bruhn A. (2017). Geo-economic variations in epidemiology, patterns of care, and outcomes in patients with acute respiratory distress syndrome: Insights from the lung safe prospective cohort study. Lancet Respir. Med..

[15] Kwon S. (2009). Thirty years of national health insurance in south korea: Lessons for achieving universal health care coverage. Health Policy Plan..

[16] Cha J.K., Oh T.K., Song I.A. (2019). Impacts of financial coverage on long-term outcome of intensive care unit survivors in south korea. Yonsei Med. J..

[17] Howard A.E., Courtney-Shapiro C., Kelso L.A., Goltz M., Morris P.E. (2004). Comparison of 3 methods of detecting acute respiratory distress syndrome: Clinical screening, chart review, and diagnostic coding. Am. J. Crit. Care.

[18] Kumar G., Taneja A., Majumdar T., Jacobs E.R., Whittle J., Nanchal R., Milwaukee Initiative in Critical Care Outcomes Research (MICCOR) Group of Investigators (2014). The association of lacking insurance with outcomes of severe sepsis: Retrospective analysis of an administrative database. Crit. Care Med..

[19] O’Brien J.M., Lu B., Ali N.A., Levine D.A., Aberegg S.K., Lemeshow S. (2011). Insurance type and sepsis-associated hospitalizations and sepsis-associated mortality among us adults: A retrospective cohort study. Crit. Care.

[20] Herridge M.S., Cheung A.M., Tansey C.M., Matte-Martyn A., Diaz-Granados N., Al-Saidi F., Cooper A.B., Guest C.B., Mazer C.D., Mehta S. (2003). One-year outcomes in survivors of the acute respiratory distress syndrome. N. Engl. J. Med..

[21] Herridge M.S., Tansey C.M., Matte A., Tomlinson G., Diaz-Granados N., Cooper A., Guest C.B., Mazer C.D., Mehta S., Stewart T.E. (2011). Functional disability 5 years after acute respiratory distress syndrome. N. Engl. J. Med..

[22] Herridge M.S., Chu L.M., Matte A., Tomlinson G., Chan L., Thomas C., Friedrich J.O., Mehta S., Lamontagne F., Levasseur M. (2016). The recover program: Disability risk groups and 1-year outcome after 7 or more days of mechanical ventilation. Am. J. Respir. Crit. Care Med..

[23] Kamdar B.B., Huang M., Dinglas V.D., Colantuoni E., Von Wachter T.M., Hopkins R.O., Needham D.M., National Heart, Lung, and Blood Institute Acute Respiratory Distress Syndrome Network (2017). Joblessness and lost earnings after acute respiratory distress syndrome in a 1-year national multicenter study. Am. J. Respir. Crit. Care Med..

[24] Kamdar B.B., Sepulveda K.A., Chong A., Lord R.K., Dinglas V.D., Mendez-Tellez P.A., Shanholtz C., Colantuoni E., Von Wachter T.M., Pronovost P.J. (2018). Return to work and lost earnings after acute respiratory distress syndrome: A 5-year prospective, longitudinal study of long-term survivors. Thorax.

[25] Fowler R.A., Noyahr L.A., Thornton J.D., Pinto R., Kahn J.M., Adhikari N.K., Dodek P.M., Khan N.A., Kalb T., Hill A. (2010). An official american thoracic society systematic review: The association between health insurance status and access, care delivery, and outcomes for patients who are critically ill. Am. J. Respir. Crit. Care Med..

